# Depression subtype classification from social media posts: few-shot prompting vs. fine-tuning of large language models

**DOI:** 10.3389/fdgth.2026.1790533

**Published:** 2026-03-23

**Authors:** Rawan AlSaad, Sulaiman Alshakhs, Rajat Thomas

**Affiliations:** 1 Weill Cornell Medicine-Qatar, Doha, Qatar; 2 Mental Health Services, Hamad Medical Corporation, Doha, Qatar

**Keywords:** artificial intelligence, depression, large language models, mental health, social media, transformers

## Abstract

**Background:**

Social media provides timely proxy signals of mental health, but reliable tweet-level classification of depression subtypes remains challenging due to short, noisy text, overlapping symptomatology, and labeling bias. Large language models (LLMs) are increasingly used in mental health for tasks such as symptom extraction, risk screening, and triage, yet their reliability for fine-grained depression subtype classification from brief social media posts remains underexplored.

**Objective:**

We benchmarked few-shot, prompt-only LLMs against parameter-efficient fine-tuned encoders for identifying depression subtypes in posts on X (formerly Twitter).

**Methods:**

We used a curated dataset of 14,983 English-language tweets stratified into six clinically grounded categories: five depression subtypes (postpartum, major, bipolar, psychotic, atypical) and a no-depression class. We compared (i) instruction-tuned causal LLMs in a few-shot setting and (ii) supervised fine-tuning of transformer encoders (e.g., RoBERTa, DeBERTa, BERTweet) under identical splits and metrics. The primary evaluation metric was macro-F1 (with accuracy, precision, recall as secondary). We also report per-class precision, recall, and F1 scores, along with confusion matrices, for the best-performing model from each model family.

**Results:**

Few-shot LLMs achieved macro-F1 = 0.73–0.77 (best: Llama-3-8B, accuracy 0.75). Fine-tuned encoders consistently outperformed prompt-only models, reaching macro-F1 = 0.94–0.96 (best: RoBERTa-large, accuracy 0.954). Relative improvements were largest for the clinically challenging classes. Fine-tuning increased F1 for postpartum and psychotic subtypes to ≈0.99 (substantially above few-shot) and boosted major-depression recall from ≈0.53–0.60 to ≈0.95–0.97. Error analyses showed prompt-only models frequently misclassified major and atypical depression as bipolar, patterns substantially reduced by fine-tuning.

**Conclusions:**

On tweet-level depression subtyping, task-specific adaptation via fine-tuning yields substantially higher and more stable performance than few-shot prompting, particularly for nuanced, clinically anchored classes. These findings recommend fine-tuned encoders as strong, compute-efficient baselines for depression subtype classification from social media.

## 1 Introduction

Mental health disorders represent a major global burden, and social media platforms such as X (formerly Twitter) provide valuable insights into individual experiences and collective discourse (1–3). Mining this data for early signals of mental health conditions offers potential for scalable surveillance, intervention design, and stigma reduction. However, classification tasks in this space are inherently complex due to noisy language, short text length, and overlapping symptomatology across conditions.

Depression is a term that encompasses several types depending on the symptoms that patients manifest and the context that they have these symptoms in. These subtypes carry distinct risk profiles, functional burdens, and treatment implications, and management strategies often depend on the specific type of depressive episode (4). Differentiating between these presentations can be challenging even for experienced clinicians (5, 6). Consequently, tools that flag nuanced patterns of depressive symptoms outside the clinical setting, for example in social media narratives, may help support more accurate diagnosis and triage and can complement rather than replace clinical judgment.

Traditional approaches have relied on supervised classifiers using bag-of-words or word embeddings, but recent advances in deep learning have introduced transformer-based models pretrained on large text corpora. Specifically, encoder-only models such as BERTweet (7) and Twitter-RoBERTa (8), trained directly on millions of tweets, represent strong baselines for social media text classification. Concurrently, instruction-tuned large language models (LLMs) such as Llama-3, Mistral, and Qwen have demonstrated impressive generalization in zero- and few-shot settings. Their ability to perform classification without explicit fine-tuning makes them attractive for low-resource clinical natural language processing (NLP) tasks. Yet, their effectiveness relative to domain-specific fine-tuning remains underexplored in healthcare contexts.

Recent Twitter/X studies increasingly adopt transformer-based models for depression detection. On English tweets, DEPTWEET formalized severity labels and showed BERT/DistilBERT baselines outperform classical methods (9). Symptom-focused work leveraged multitask BERT with Patient Health Questionnaire-9 (PHQ-9) guidance to improve robustness to figurative language at tweet level (10). In Arabic Twitter, AraDepSu reported MARBERT as the top encoder among 30+ transformer variants on a three-class tweet task (depressed/suicidal/neutral) (11). Yet generalization remains fragile: an NAACL 2024 study (12) auditing cross-country generalization in depression detection found sizable performance drops even for strong pretrained models. User-level identification with fine-tuned DistilBERT demonstrates feasibility but modest discrimination, underscoring the need for better supervision and domain transfer (13). However, prior work is largely binary or severity-oriented, with little tweet-level multi-class subtype classification. A notable exception is Nusrat et al. (14), who developed a tweet-level pipeline that applied lexicon-derived labels to five depression subtypes and trained BERT-based classifiers.

Building on previous literature that developed tweet-level pipelines aligned with several depression subtypes, our study systematically benchmarks both paradigms—prompt-only LLM classification and supervised fine-tuning with LoRA adapters—on a curated mental-health tweet dataset, using identical splits and metrics and per-subtype error analyses. Our benchmark provides practical guidance on when prompt-only models suffice and when task-specific fine-tuning is required for reliable, clinically nuanced tweet-level depression subtyping.

## 2 Methods

### 2.1 Dataset and task definition

**Figure 1:**
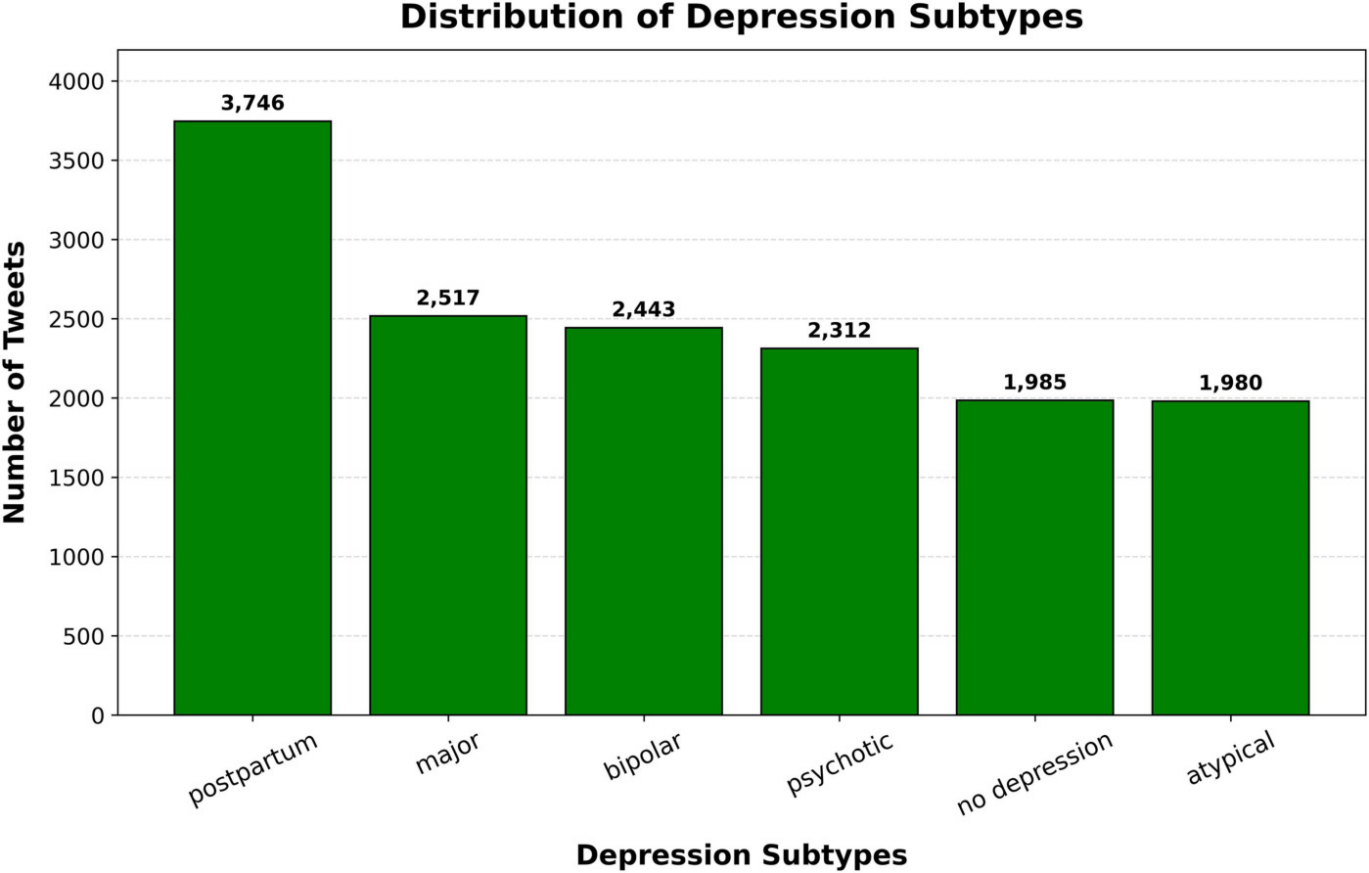
Distribution of depression subtypes in the dataset.

We use a curated, tweet-level, single-label multiclass dataset for depression subtype classification (15). The corpus contains 14,983 English-language tweets annotated into six mutually exclusive categories: postpartum depression (24.9%), major depression (16.8%), bipolar depression (16.3%), psychotic depression (15.4%), atypical depression (13.2%), and no depression (13.2%) (Figure 1). Major depressive disorder (MDD) is an umbrella term for symptoms of depression that are severe enough to be diagnosed as a clinical disorder. The Diagnostic and Statistical Manual of Mental Disorders, 5th Edition (DSM-5) (16) denotes MDD episodes with persistent low mood and/or anhedonia with neurovegetative changes (e.g., sleep, appetite, concentration) and functional impairment, among other symptoms. To be diagnosed with MDD, patients should not have had a history of a manic or hypomanic episode. The DSM-5 has several specifiers for MDD depending on the prevailing symptoms. For the purposes of this dataset, MDD with psychotic features is categorized as “psychotic depression”, which reflects hallucinations, delusions, or marked disorganization during a depressive episode. MDD with atypical features such as mood reactivity, hypersomnia, hyperphagia/weight gain, leaden paralysis, and interpersonal rejection sensitivity is categorized as “atypical depression”. MDD with all other possible specifiers—other than with peripartum onset—is categorized as “major depression”. The original dataset had another category for “postpartum depression”, which refers to MDD that emerges in the first year after childbirth (17). People with bipolar disorder are characterized by having episodes of mania/hypomania alternating with depressive episodes. Depressive episodes in the context of bipolar disorder are categorized as “bipolar depression” (16). The last category is lack of any form of depressive episode, characterized as “no depression”. It is important to note that subtype labels in this dataset are derived from content-based annotations of individual posts rather than formal clinical diagnoses; therefore, they should be interpreted as linguistic proxies for subtype-related expressions rather than confirmed psychiatric classifications.

We cast depression subtype identification as a six-way, single-label classification task: each tweet $\;x_{i}$ receives exactly one label *y_i_* ∈ {postpartum, major, bipolar, psychotic, atypical, no depression}.

### 2.2 Model families and protocols

We compared two families of methods for tweet-level depression subtype classification: (i) prompt-only, instruction-tuned LLMs evaluated in a few-shot regime without parameter updates, and (ii) fine-tuned transformer encoders adapted with low-rank adapters (LoRA) for efficient supervised learning. In total, we benchmarked 14 models: 5 prompt-only LLMs and 9 fine-tuned encoders. Together, these two tracks: prompt-only and LoRA fine-tuning, allow us to contrast in-context learning against parameter-efficient supervised adaptation under identical data splits and evaluation protocols. We applied minimal preprocessing (standard normalization and tokenizer processing) and did not implement explicit rules for negation (e.g., “not depressed”) or sarcasm detection. Given that both LLMs and transformer encoders model contextual semantics, we kept inputs consistent to avoid confounding model comparisons with handcrafted linguistic filters.

#### 2.2.1 Prompt-only instruction-tuned LLMs

We evaluate instruction-tuned causal LLMs using a fixed multiple-choice prompt that requires selecting exactly one label from the six clinically defined categories. The prompt contains: (i) a brief instruction; (ii) six labeled options (A–F) corresponding to {postpartum, major, bipolar, psychotic, no depression, atypical}; (iii) the target tweet; and (iv) one few-shot exemplar per class drawn once from the training split using a fixed random seed, preferring short, clean tweets (no URLs/@mentions). Models are instructed to return a single letter.

For robustness, we use self-consistency voting with k=5 independent prompt realizations per tweet. Each realization yields next-token probabilities for the option letters; we score letters by their log-probability under the model's native tokenizer and take the majority vote, mapping the chosen letter back to its class. This pipeline performs no weight updates and keeps all hyperparameters fixed across models.

Using this shared protocol, we benchmark five open, compact instruction-tuned decoders: Meta-Llama-3-8B-Instruct, Mistral-7B-Instruct-v0.2, Phi-3.5-mini-instruct, Qwen2.5-7B-Instruct, and Gemma-2-2B-it. This selection spans 2–8B parameters and diverse pretraining corpora, tokenizers, and alignment recipes, allowing us to test whether prompt-only performance is consistent across families rather than driven by a single model's inductive biases. All models expose next-token probabilities required by our scoring. The specific checkpoints are listed in Table 1.

**Table 1:** Model families and specifications.

Few-shot Prompting (instruction-tuned decoder LLMs)			
#	Model	Model type	Model size*
1	Meta-llama/Meta-Llama-3-8B-Instruct	Decoder-only (causal LM)	8 B
2	Mistralai/Mistral-7B-Instruct-v0.2	Decoder-only (causal LM)	7.3 B
3	Microsoft/Phi-3.5-mini-instruct	Decoder-only (causal LM)	3.8 B
4	Qwen/Qwen2.5-7B-Instruct	Decoder-only (causal LM)	7 B
5	Google/gemma-2-2b-it	Decoder-only (causal LM)	2 B
Fine-Tuning (encoder backbones for supervised tasks)			
	Model	Model type	Model size
6	Microsoft/deberta-v3-large	Encoder (MLM)	∼418 M (24 × 1,024; 128 K vocab)
7	Microsoft/deberta-v2-xlarge	Encoder (MLM)	∼900 M (24 × 1,536)
8	Vinai/bertweet-large	Encoder (RoBERTa-style MLM)	∼355 M
9	Vinai/bertweet-base	Encoder (RoBERTa-style MLM)	∼135 M
10	Cardiffnlp/twitter-roberta-base	Encoder (RoBERTa-base)	∼125 M
11	Roberta-large	Encoder (RoBERTa-large)	∼355 M
12	Cardiffnlp/twitter-xlm-roberta-base	Encoder (XLM-R base)	∼270 M
13	Microsoft/mpnet-base	Encoder (MPNet)	∼110 M
14	Albert-xxlarge-v2	Encoder (ALBERT)	∼223 M (parameter sharing)

Few-shot prompting uses instruction-tuned decoder LLMs; fine-tuning uses encoder backbones adapted for supervised classification. Model size is the number of trainable parameters (*approximate).

#### 2.2.2 Parameter-efficient fine-tuning of encoders

We fine-tune transformer encoders for sequence classification using LoRA, injecting small trainable rank-decomposed adapters into selected projection layers while freezing the base weights. LoRA substantially reduces trainable parameters and memory while preserving encoder capacity. For DeBERTa variants, LoRA is attached to attention projections (query_proj, key_proj, value_proj, dense); for other encoders, to query, key, value, and feed-forward projections. Unless specified, we set rank r=8,α=16,dropout=0.05. Inputs use each model's native tokenizer with truncation; the effective maximum length is 160 tokens (including special tokens), respecting each model's positional limit.

Optimization uses AdamW, learning rate $2\times 10^{-4}$ (applied to the classifier head and LoRA parameters), weight decay 0.03, a cosine schedule with warm-up ratio 0.06, and gradient clipping = 1.0; batch size = 16, up to 10 epochs with early stopping on validation macro-F1 (patience = 2, improvement threshold $5\times 10^{-4}$).

We fine-tune nine encoders spanning architecture families, scales, and pretraining regimes: DeBERTa-v3-large, DeBERTa-v2-xlarge, RoBERTa-large, BERTweet-large/base, cardiffnlp/twitter-roberta-base, MPNet-base, cardiffnlp/twitter-xlm-roberta-base, and ALBERT-xxlarge-v2. This balances domain-specific and general encoders (∼110M–900M parameters) and distinct pretraining objectives, enabling a fair comparison to prompt-only LLMs under identical splits and metrics.

### 2.3 Training objective

$\mathrm{Let}\;\{(x_{i},y_{i}){\}}_{i=1}^{N}$ denote *N* tweet-label pairs, $y_{i}\in \{1,\ldots ,C\},\;C=\;6$. Denote logits $z_{i}\in R^{C}$, probabilities $\;p_{i}=\mathrm{softmax}(z_{i})$, and class counts$\;n_{c}(c=1,\ldots ,C)$. Our objective combines class-balanced focal loss, LDAM margins, R-Drop consistency, and a targeted pairwise margin penalty:

1. **Class-balanced focal with LDAM.** We reweight classes using the class-balanced estimator and apply Label-Distribution-Aware Margin (LDAM) by subtracting a class-dependent margin from the true-class logit, then optimize focal cross-entropy on the margin-shifted logits (up-weighting hard/minority cases). We define class weights:
  $$w_{c}=\frac{\frac{\left(1-\beta \right)}{1-\beta^{n_{c}}}}{\frac{1}{C}\sum_{\;j=1}^{C}\frac{\left(1-\beta \right)}{1-\beta^{n_{j}}}},\;\beta =0.999;\;m_{c}=\frac{C_{0}}{n_{c}^{1/4}},\;C_{0}=0.5;$$
  $${\tilde{z}}_{i,y_{i}}=z_{i,y_{i}}-m_{y_{i}},\;{\tilde{z}}_{i,k}=z_{i,k}(k\neq y_{i})$$

The focal term is:

$$L_{\mathrm{CB}\text{-}\mathrm{Focal}\;+\;\mathrm{LDAM}}=-w_{y_{i}}(1-p_{i,y_{i}}{)}^{\gamma }\;\log\;p_{i,y_{i}},\;\mathrm{with}\;\gamma =2.0,\;p_{i}=\mathrm{softmax}(\tilde{z_{i}}).$$

2. **R-Drop (symmetric KL).** We use R-Drop as a consistency regularizer: for the same input, we run the model twice with different dropout masks to obtain two predictive distributions $p_{i}^{\left(1\right)}\;\mathrm{and}\;p_{i}^{\left(2\right)}$. We then penalize their discrepancy with a symmetric KL divergence, encouraging the network to produce stable predictions under stochastic perturbations (e.g., dropout/LoRA-dropout). This reduces overfitting and improves calibration without changing the main supervision signal.

For two stochastic forward passes $z_{i}^{\left(1\right)},z_{i}^{\left(2\right)}$ with shared inputs,

$$L_{RDop}=\frac{1}{2}[KL(\;p_{i}^{\left(1\right)}||\;p_{i}^{\left(2\right)})+KL(\;p_{i}^{\left(2\right)}||\;p_{i}^{\left(1\right)})],\;\mathrm{where}\;p_{i}^{\left(k\right)}=\mathrm{softmax}(z_{i}^{\left(k\right)})$$

3. **Pairwise margin penalty (targeted).** For $y_{i}=\mathrm{bipolar}$, encourage a margin over “no depression” and “atypical”:
  $$L_{\mathrm{pair}}=\max(0,\delta -(z_{i,\mathrm{bipolar}}-z_{i,\mathrm{no}}))+\max(0,\delta -(z_{i,\mathrm{bipolar}}-z_{i,\mathrm{atypical}})),\;\delta =\;0.15.$$

The final loss for each batch is

$$\begin{array}{cc} L\; & \underset{\mathrm{stochastic}\;\mathrm{passes}}{\underbrace{=\frac{1}{2}(L_{\mathrm{CB}0\mathrm{Focal}\;+\;\mathrm{LDAM}}^{\left(1\right)}+L_{\mathrm{CB}0\mathrm{Focal}\;+\;\mathrm{LDAM}}^{\left(2\right)})}}+\lambda_{\mathrm{rd}}L_{RDrop}\;\\ \; & +\lambda_{\mathrm{pair}}L_{\mathrm{pair}\;},\;\;\\ \; & \lambda_{\mathrm{rd}}=0.5,\;\;\lambda_{\mathrm{pair}}=0.15. \end{array}$$

Class imbalance was handled at the loss level without oversampling or undersampling, using class-balanced focal weighting and LDAM to adapt the optimization signal to class frequency differences.

### 2.4 Evaluation setup

Our primary metric is macro-F1; we also report accuracy, precision, and recall. For completeness, we report per-class precision, recall, and F1 scores, along with confusion matrices, on the test set for the best-performing model from each model family. Prompt-only LLMs and fine-tuned encoders are evaluated on exactly the same splits and tweets. For the prompt-only track we decode the majority letter from the self-consistency votes (k = 5) and map it to the corresponding class; for the fine-tuned track we decode the argmax over class logits (or averaged logits when MC-dropout is used). Because the dataset does not provide user IDs or timestamps, we used the stratified train/validation/test splits and additionally ensured there were no exact-duplicate tweet texts shared across partitions (to minimize content leakage). Computation was performed on a single NVIDIA A100 (80 GB) GPU.

## 3 Results

### 3.1 Overall performance

Across 14 models, fine-tuned encoders substantially outperformed few-shot, prompt-only LLMs on tweet-level subtype classification (6 classes) (Tables 2, 3). The best prompt-only models Llama-3-8B-Instruct and Mistral-7B-Instruct each reached macro-F1 0.765 (Acc 0.750 and 0.757, respectively), while Phi-3.5-mini, Qwen2.5-7B, and Gemma-2-2B-it clustered at macro-F1 0.733–0.739 (Acc 0.728–0.745). In contrast, the top encoders achieved macro-F1 0.956–0.957. All nine encoders were strong, with the lowest at macro-F1 0.938 (ALBERT-xxlarge) and the highest at 0.957 (RoBERTa-large), reinforcing the fine-tuning advantage even for smaller backbones. Validation and test tracked closely for each model (e.g., RoBERTa-large: Val macro-F1 0.951 vs. Test 0.957).

**Table 2:** Performance of few-shot prompting and fine-tuned models on the validation and test sets, evaluated using macro-F1 and accuracy.

#	Model	Type	Val macro-F1	Val Acc	Test macro-F1	Test Acc
1	Meta-Llama-3-8B-Instruct	Few-shot	0.774	0.758	**0** **.** **765**	0.750
2	Mistral-7B-Instruct-v0.2	Few-shot	0.769	0.757	**0** **.** **765**	**0** **.** **757**
3	Phi-3.5-mini-instruct	Few-shot	0.741	0.731	0.738	0.730
4	Qwen2.5-7B-Instruct	Few-shot	0.744	0.732	0.739	0.728
5	Gemma-2-2B-it	Few-shot	0.743	0.751	0.733	0.745
6	DeBERTa-v3-large	Fine-tune	0.950	0.947	0.956	0.953
7	DeBERTa-v2-xlarge	Fine-tune	0.952	0.949	0.953	0.950
8	BERTweet-large	Fine-tune	0.951	0.949	0.956	0.953
9	BERTweet-base	Fine-tune	0.943	0.939	0.948	**0** **.** **945**
10	Twitter-RoBERTa-base	Fine-tune	0.940	0.937	0.950	0.948
11	RoBERTa-large	Fine-tune	0.951	0.948	**0** **.** **957**	0.954
12	Twitter-XLM-RoBERTa-base	Fine-tune	0.940	0.937	0.947	0.944
13	MPNet-base	Fine-tune	0.942	0.938	0.944	0.941
14	ALBERT-xxlarge-v2	Fine-tune	0.932	0.928	0.938	0.933

Bold values indicate the best-performing result(s) for each metric.

**Table 3:** Performance of few-shot prompting and fine-tuned models on the validation and test sets, evaluated using precision and recall.

#	Model	Val Precision	Val Recall	Test Precision	Test Recall
1	Meta-Llama-3-8B-Instruct	0.860	0.775	**0** **.** **853**	0.768
2	Mistral-7B-Instruct-v0.2	0.821	0.778	0.817	**0** **.** **775**
3	Phi-3.5-mini-instruct	0.813	0.742	0.809	0.740
4	Qwen2.5-7B-Instruct	0.811	0.748	0.805	0.736
5	Gemma-2-2B-it	0.824	0.744	0.836	0.733
6	DeBERTa-v3-large	0.950	0.950	**0** **.** **957**	**0** **.** **955**
7	DeBERTa-v2-xlarge	0.952	0.952	0.953	0.952
8	BERTweet-large	0.952	0.951	0.955	0.952
9	BERTweet-base	0.944	0.942	0.946	0.944
10	Twitter-RoBERTa-base	0.942	0.939	0.948	0.945
11	RoBERTa-large	0.952	0.950	0.956	0.952
12	Twitter-XLM-RoBERTa-base	0.944	0.938	0.944	0.947
13	MPNet-base	0.944	0.941	0.949	0.943
14	ALBERT-xxlarge-v2	0.933	0.933	0.937	0.938

Bold values indicate the best-performing result(s) for each metric.

Across approach families, the best fine-tuned encoder (RoBERTa-large) outperformed the best few-shot, prompt-only model (Llama-3-8B) by +19.2 percentage points in macro-F1 and +20.4 percentage points in accuracy (0.957/0.954 vs. 0.765/0.750), indicating a large, consistent advantage for fine-tuning over few-shot prompting.

### 3.2 Depression class-specific analysis

**Figure 2:**
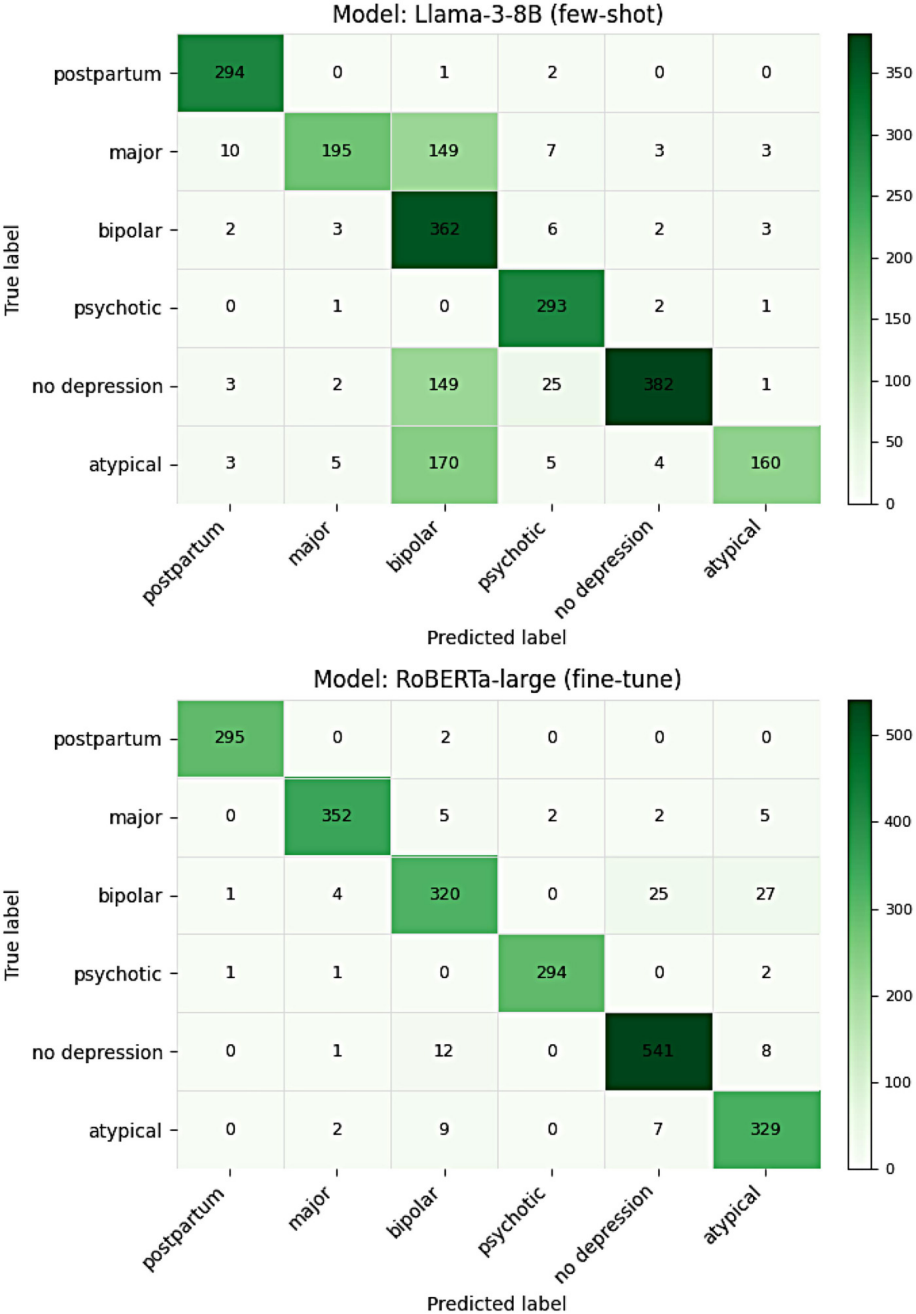
Confusion matrices on the test set for the best prompt-only model (llama-3-8B, few-shot; top) and the best fine-tuned encoder (RoBERTa-large; bottom).

Using the strongest prompt-only LLM, Meta-Llama-3-8B, bipolar shows high recall (362/378 = 95.8%) but low precision (362/831 = 43.6%) due to substantial misclassification from no depression (149/562 = 26.5%) and atypical (170/347 = 49.0%); major depression recall is 53.1% (195/367), while postpartum and psychotic are near-perfect (99.0% and 98.7%), and no depression recall is 68.0% (382/562) (Figure 2).

For the top encoder (RoBERTa-large), *bipolar* precision improves (325/350 = 92.9%) with fewer cases of *no depression* misclassified as *bipolar* (8/562 = 1.4%), while *bipolar* recall is 325/378 = 86.0%, with misses split mainly into *no depression* (28/378 = 7.4%) and *atypical* (22/378 = 5.8%). *Major depression* remains high (355/367 = 96.7%); *postpartum* and *psychotic* are near-perfect (99.7%, 99.7%); and *no depression* recall is 97.0% (Figure 2).

## 4 Discussion

### 4.1 Main findings

This study shows that parameter-efficient fine-tuning of encoder models consistently outperforms few-shot prompting for tweet-level depression subtype classification. The best fine-tuned encoder (RoBERTa-large) exceeds the best prompt-only model (Llama-3-8B) by Δmacro-F1 = +0.192 and ΔAccuracy = +0.204 on the same test set. Fine-tuned encoders nearly saturate postpartum and psychotic (F1 ≈ 0.99) and substantially improve major depression recall, while the prompt-only family exhibits concentrated errors where major depression and atypical are misclassified as bipolar. Especially with atypical depression, the difficulty in distinguishing it from bipolar may be understandable given the association between bipolar type 2 and atypical symptoms of depression (18). Performance is consistent across encoder backbones (macro-F1 0.938–0.957).

Non-diagnostic, clinician-in-the-loop flagging of high-risk subtypes-related linguistic signals appears feasible with our fine-tuned encoders, under this dataset's labeling regime. In particular, near-saturated performance for postpartum and psychotic classes (F1 ≈ 0.99) and markedly higher recall for major depression (≈0.95–0.97) suggest the model can reduce missed cases while keeping false positive bipolar flags low relative to prompt-only baselines.

While the empirical performance advantage of fine-tuned encoders over prompt-only LLMs is robust across metrics, the clinical interpretation of this gap should be made cautiously. Given that labels are proxy annotations derived from short posts rather than clinician-administered assessments, improved performance may reflect enhanced sensitivity to dataset-specific lexical regularities rather than deeper subtype reasoning. Accordingly, our primary contribution is a controlled comparison of modeling paradigms under a consistent labeling regime, rather than a claim of clinically validated subtype detection. Therefore, all performance and error analyses should be primarily interpreted as evidence of improved discrimination of subtype-related linguistic patterns within this benchmark, rather than as demonstration of clinically validated subtype classification.

### 4.2 Research and clinical implications

For targeted, multi-class clinical phenotyping on short social-media text, learning task-specific decision boundaries matters more than in-context reasoning alone. LoRA fine-tuning is compute-light and reproducible, making it a pragmatic choice for research groups without access to very large models. The error reductions on bipolar, particularly fewer false positives from no depression and atypical, are important for downstream screening pipelines where over-flagging can erode trust and waste clinical review time. More broadly, the results caution against relying on generic few-shot prompting when the objective requires fine-grained, clinically anchored distinctions at tweet level.

Translationally, two near-term directions emerge. First, deploy subtype-aware surveillance to estimate trajectories for major vs. psychotic depression signals, especially during external shocks (pandemics, policy shifts), with uncertainty bands and drift monitoring. Second, aggregate from tweet- to user/session-level phenotypes using temporal smoothing to stabilize signals, curb spurious flags, and better prioritize human review and tailored signposting in opt-in programs.

Clinically, flagging patient social media data into tools may be of value to diagnosis and management in multiple situations. For instance, there are challenges which may lead toward mislabeling bipolar depression as major depression. This can be of detriment to the plan of care as managing depression with the standard pharmacological option of an antidepressant can trigger a manic episode in someone who has undiscovered bipolar disorder and is not on mood stabilizing (5). Therefore, having historical data which suggest the presence of mania or hypomania prior to the depressive episode is especially vital here. Another situation where such tools may assist, albeit with an even less proneness to errors, is when the flags include the script of the tweet that they were based on. That way, the clinician can synthesize the actual flagged data along with the patient interview to generate a proper assessment and plan. A third implication for these tools is in risk stratification, where patients’ expression of themselves through social media can be used to screen for symptoms of depression which if left untreated can cause the patient to be risky to themselves or others, depending on the surrounding circumstances and type of depression these patients have.

When the topic of surveilling the social media activity of patients comes up, it becomes of need to discuss the ethical implications on two important fronts. First, the act of surveilling patients’ activity outside of the clinical setting, especially by non-human technological systems, is a new field. One area that needs to be studied is how it affects the activity of patients knowing that they are monitored (19). Another important aspect is the possibility of inadvertently jeopardizing patients’ autonomy. To counteract that, there must be a focus on patient consent to have this data monitored but equally importantly is the stress on the optionality to opt in. Although patients who seek mental health services do generally share sensitive information with professionals, it is often limited by what the patients choose to bring to the interview room. Therefore, care and sensitivity should be utilized when navigating the offering of such services to patients (20). Beyond concerns related to privacy, surveillance, and informed consent, it is important to consider the potential misuse or overinterpretation of algorithmically generated diagnostic labels. Automated classification of depression subtypes based on social media language could be misapplied in contexts such as employment screening, insurance risk profiling, or other non-clinical decision-making, raising risks of stigma and discrimination if safeguards are not in place. Additionally, awareness among users that their online expressions may be monitored or analyzed for mental health inference could alter natural communication patterns, potentially leading to self-censorship or changes in help-seeking behavior. These considerations underscore the need for transparent governance frameworks, clear communication about intended use, and strict limitations ensuring that such systems are deployed only as supportive tools under appropriate ethical and clinical oversight.

Consistent with prior literature (21), our study shares several known challenges in social-media mental health modeling, including reliance on weakly supervised annotations, potential domain-specific lexical cues, and uncertainty in cross-platform generalizability. To further examine whether subtype discrimination was driven by trivial lexical cues or annotation artifacts, we conducted a *post-hoc* qualitative assessment comparing predictions from the best prompt-only model (Llama-3-8B) and the best fine-tuned encoder (RoBERTa-large). Representative examples and analysis are provided in Supplementary Material. Generalizability beyond Twitter remains an important consideration. Other platforms (e.g., Reddit, forums, or longer-form posts) differ in text length, conversational structure, and user demographics, which can introduce domain shift and alter the distribution of depression-related language cues. Consequently, performance may attenuate without adaptation, and external validation on multi-platform corpora is warranted. Future work should evaluate cross-platform transfer and consider lightweight domain adaptation (e.g., continued pretraining or parameter-efficient fine-tuning) to improve robustness.

Together, these directions aim to translate robust tweet-level classifiers into safer, more generalizable, and operational mental-health monitoring workflows that support clinician oversight and perform reliably across populations and platforms.

### 4.3 Limitations

First, a key limitation relates to the validity and granularity of subtype labels derived from single social media posts. While the dataset is described as clinically grounded, annotations are necessarily based on observable linguistic cues rather than longitudinal clinical assessment, and thus may reflect expressions consistent with a subtype rather than true diagnostic categorization. The near-ceiling performance observed for postpartum and psychotic depression likely reflects the presence of lexically salient indicators (e.g., references to childbirth or hallucinations) that are easier to detect algorithmically. In contrast, the confusion observed among major, atypical, and bipolar depression may be influenced not only by model limitations but also by overlapping symptom language, label ambiguity, and the inherently weak supervision of tweet-level annotations. Accordingly, results should be interpreted as reflecting the models’ ability to distinguish patterns of subtype-related discourse rather than making clinical diagnostic distinctions. Future work would benefit from datasets incorporating clinician-verified labels, longitudinal context, or multimodal clinical information to better assess subtype reasoning. Relatedly, models may also benefit from shortcut learning, where predictions rely on spurious lexical artifacts or platform-specific cues correlated with particular labels. This risk may be higher for classes with highly distinctive vocabulary, and it motivates robustness-oriented evaluation beyond in-domain test sets. Future work should therefore include cross-platform and cross-temporal validation, as well as counterfactual/adversarial tests (e.g., vocabulary swaps or cue masking) to quantify reliance on surface-form signals and to assess whether gains persist under reduced lexical shortcuts. Second, the study is limited to English and to single-tweet classification; we did not evaluate user-level context, longitudinal patterns, or cross-lingual generalization. Third, external validity is uncertain: data come from a single platform and time window, so distribution shifts (e.g., policy changes) or demographic skews could degrade performance, and subgroup fairness audits were not possible (e.g., by gender, age, or geography). Fourth, our label space is constrained by the original dataset's six depression subtypes, which do not capture comorbidity, symptom dynamics, or other prevalent depressive disorders such as dysthymia.

## 5 Conclusion

This benchmark demonstrates that parameter-efficient, fine-tuned encoders deliver markedly higher and more stable performance than few-shot, prompt-only LLMs for tweet-level depression subtyping. On the same test set, fine-tuned RoBERTa-large achieved macro-F1 0.957 (accuracy 0.954) vs. 0.765 (0.750) for the best prompt-only model, with near-saturated F1 for postpartum and psychotic classes and large gains in major-depression recall. These findings establish fine-tuned encoders as strong, compute-efficient baselines for social-media mental-health NLP. Clinically, models should be deployed only as non-diagnostic, clinician-in-the-loop screeners with conservative thresholds, calibration, and drift monitoring. Future work should evaluate user-level aggregation, multilingual transfer, and fairness, and test prospective utility in opt-in settings such as helplines and perinatal programs.

## Data Availability

The data used in this study are publicly available and can be accessed through the Zenodo repository at https://doi.org/10.5281/zenodo.14233292.

## Code availability

The code used for analysis is publicly available at https://github.com/rawan-alsaad/depression-subtype-llm-benchmark.

## References

[1] Kim J Uddin ZA Lee Y Nasri F Gill H Subramanieapillai M A systematic review of the validity of screening depression through Facebook, Twitter, Instagram, and Snapchat. J Affect Disord. (2021) 286:360–9. 10.1016/j.jad.2020.08.091 33691948

[2] Safa R Bayat P Moghtader L. Automatic detection of depression symptoms in twitter using multimodal analysis. J Supercomput. (2022) 78(4):4709–44. 10.1007/s11227-021-04040-8 34518741 PMC8426595

[3] Helmy A Nassar R Ramdan N. Depression detection for twitter users using sentiment analysis in English and Arabic tweets. Artif Intell Med. (2024) 147:102716. 10.1016/j.artmed.2023.102716 38184345

[4] Bowden CL Singh V. The use of antidepressants in bipolar disorder patients with depression. Expert Opin Pharmacother. (2016) 17(1):17–25. 10.1517/14656566.2016.1104299 26479314

[5] Phillips ML Kupfer DJ. Bipolar disorder diagnosis: challenges and future directions. Lancet. (2013) 381(9878):1663–71. 10.1016/S0140-6736(13)60989-7 23663952 PMC5858935

[6] Rothschild AJ Mulsant BH Meyers BS Flint AJ. Challenges in Differentiating and Diagnosing. US: SLACK (2006). p. 40–6.

[7] Nguyen DQ Vu T Nguyen AT. BERTweet: A pre-trained language model for English Tweets. *arXiv* [Preprint]. *arXiv:2005.10200*. (2020). Available online at: https://arxiv.org/abs/2005.10200 (Accessed December 29, 2025).

[8] Zaman A Ferdous SS Akhter N Tagore T Nabi MM Ali KMA. DARN: dual-attention RoBERTa network for depression severity detection from twitter. 2023 26th International Conference on Computer and Information Technology (ICCIT); (2023).

[9] Kabir M Ahmed T Hasan MB Laskar MTR Joarder TK Mahmud H DEPTWEET: a typology for social media texts to detect depression severities. Comput Human Behav. (2023) 139:107503. 10.1016/j.chb.2022.107503

[10] Yadav S Chauhan J Sain JP Thirunarayan K Sheth A Schumm J. Identifying depressive symptoms from Tweets: figurative language enabled multitask learning framework. In Proceedings of the 28th International Conference on Computational Linguistics. Barcelona, Spain: International Committee on Computational Linguistics (2020). p. 696–709.

[11] Hassib M Hossam N Sameh J Torki M. AraDepSu: detecting depression and suicidal ideation in Arabic Tweets using transformers. In Proceedings of the Seventh Arabic Natural Language Processing Workshop (WANLP). Abu Dhabi, United Arab Emirates (Hybrid): Association for Computational Linguistics (2022). p. 302–311.

[12] Abdelkadir NA Zhang C Mayo N Chancellor S. Diverse perspectives, divergent models: cross-cultural evaluation of depression detection on Twitter. InProceedings of the 2024 Conference of the North American Chapter of the Association for Computational Linguistics: Human Language Technologies (Volume 2: Short Papers). Mexico City, Mexico: Association for Computational Linguistics (2024). p. 672–80.

[13] Adarlo MA Leon MD. Detecting potential depressed users in twitter using a fine-tuned DistilBERT model. In: Ahram T Kalra J Karwowski W, editors. Artificial Intelligence and Social Computing. New York: AHFE Open Access (AHFE International) (2022). p. 155–63.

[14] Nusrat MO Shahzad W Jamal SA. Multi Class Depression Detection Through Tweets using Artificial Intelligence. *arXiv* [Preprint]. arXiv:2404.13104 (2024). Available online at: https://arxiv.org/abs/2404.13104 (Accessed January 03, 2025).

[15] Nusrat MO. Multi-Class Depression Detection Dataset (v1.0). (2024). Available online at: https://zenodo.org/records/14233292 (Accessed July 1, 2025).

[16] American Psychiatric Association. Diagnostic and Statistical Manual of Mental Disorders. 5th ed. American Psychiatric Association (2013).

[17] Stuart-Parrigon K Stuart S. Perinatal depression: an update and overview. Curr Psychiatry Rep. (2014) 16(9):468. 10.1007/s11920-014-0468-6 25034859 PMC4920261

[18] Łojko D Buzuk G Owecki M Ruchała M Rybakowski JK. Atypical features in depression: association with obesity and bipolar disorder. J Affect Disord. (2015) 185:76–80. 10.1016/j.jad.2015.06.020 26148463

[19] Aiello AE Renson A Zivich PN. Social media- and internet-based disease surveillance for public health. Annu Rev Public Health. (2020) 41:101–18. 10.1146/annurev-publhealth-040119-094402 31905322 PMC7959655

[20] Jin Y Liu J Li P Wang B Yan Y Zhang H The applications of large language models in mental health: scoping review. J Med Internet Res. (2025) 27:e69284. 10.2196/69284 40324177 PMC12089884

[21] Cao Y Dai J Wang Z Zhang Y Shen X Liu Y Machine learning approaches for depression detection on social Media: a systematic review of biases and methodological challenges. J Behav Data Sci. (2025) 5(1):67–102. 10.35566/jbds/caoyc

