# Supplementary material for: Depression subtype classification from social media posts: few-shot prompting vs. fine-tuning of large language models
Source: Front Digit Health. 2026 Mar 23;8:1790533. doi: 10.3389/fdgth.2026.1790533 (PMC13051289; doi:10.3389/fdgth.2026.1790533)
Supplement: Supplementary file 1 [file Supplementaryfile1.docx]

# Supplementary Material A. Post-hoc Qualitative Assessment of Model Predictions

To further examine whether subtype discrimination was driven by trivial lexical cues or annotation artifacts, we conducted a post-hoc qualitative assessment comparing predictions from the best prompt-only model (Llama-3-8B) and the best fine-tuned encoder (RoBERTa-large). We manually reviewed a subset of tweets from the test set, focusing on three categories: (i) cases without explicit subtype-revealing keywords, (ii) clinically ambiguous expressions where multiple subtypes may plausibly apply, and (iii) potential annotation ambiguities or noisy labels. Representative examples illustrating these categories are summarized below.

## A.1 Representative Examples

## A.1.1 Cases Without Explicit Subtype Keywords

These tweets do not contain obvious subtype-specific keywords (e.g., “postpartum”, “psychosis”, or “bipolar”), requiring models to rely on contextual language patterns rather than simple lexical cues.

**Example 1**

Tweet:
“I barely slept again last night and my mind keeps racing like I can't shut it off.”

True label: Bipolar depression
Llama-3-8B: Major depressive
RoBERTa-large: Bipolar depression

Observation:
The tweet contains no explicit bipolar terminology. RoBERTa correctly captures contextual signals such as racing thoughts and sleep disturbance, whereas the prompt-only model interprets the language as general depressive symptoms.

**Example 2**

Tweet:
“I feel completely detached from reality lately and nothing makes sense anymore.”

True label: Psychotic depression
Llama-3-8B: Major depressive
RoBERTa-large: Psychotic depression

Observation:
The tweet does not explicitly mention hallucinations or delusions. RoBERTa identifies the severe cognitive disorganization implied in the statement, while the prompt-based model interprets it as generic depressive language.

**Example 3**

Tweet:
“Everything feels heavy and I can’t even explain why.”

True label: Major depressive
Llama-3-8B: Bipolar
RoBERTa-large: Major depressive

Observation:
The tweet contains vague emotional language. RoBERTa correctly predicts major depression, while the prompt-only model misinterprets the statement as bipolar due to ambiguous mood-related wording.

## A.1.2 Clinically Ambiguous Expressions

Some tweets contain language that overlaps across multiple depression subtypes, making classification inherently difficult even for human reviewers.

**Example 1**

Tweet:

“I feel exhausted all the time but my mind won’t stop jumping between ideas.”

True label: Bipolar depression
Llama-3-8B: Major depressive
RoBERTa-large: Bipolar depression

Observation:
The tweet combines fatigue (typical of depression) with racing thoughts (often associated with bipolar mood instability). RoBERTa appears better able to integrate these signals.

**Example 2**

Tweet:
“I cry constantly but sometimes I suddenly feel like I can do everything at once.”

True label: Bipolar depression
Llama-3-8B: Major depressive
RoBERTa-large: Bipolar depression

Observation:
The tweet mixes depressive and hypomanic cues. RoBERTa captures the alternating mood pattern characteristic of bipolar disorder.

**Example 3**

Tweet:
“I feel empty most days but occasionally I get bursts of energy that don’t last.”

True label: Bipolar depression
Llama-3-8B: Major depressive
RoBERTa-large: Major depressive

Observation:
Even the fine-tuned model struggles with ambiguous signals where short bursts of energy may reflect mood variability rather than true hypomania.

## A.1.3 Likely Annotation Ambiguities

Some disagreements appear to reflect potential ambiguity or noise in tweet-level annotations rather than clear modeling errors.

**Example 1**

Tweet:
“I hate feeling like this every day.”

True label: Major depressive
Llama-3-8B: Major depressive
RoBERTa-large: Atypical

Observation:
The tweet provides minimal contextual information. Without additional context, distinguishing between major and atypical depression is difficult even for human annotators.

**Example 2**

Tweet:
“Nothing makes sense anymore and I just want to disappear.”

True label: Major depressive
Llama-3-8B: Psychotic
RoBERTa-large: Major depressive

Observation:
The language expresses severe distress but does not clearly indicate psychosis. The prompt-based model appears to over-interpret the intensity of emotional expression.

**Example 3**

Tweet:
“Everything is exhausting lately.”

True label: Atypical depression
Llama-3-8B: Major depressive
RoBERTa-large: Major depressive

Observation:
The tweet lacks clear indicators distinguishing atypical from major depression, suggesting that some annotation decisions may rely on contextual interpretation beyond the tweet itself.

## A.2 Observations from Qualitative Review

Although some depression subtypes in social media data are associated with recognizable lexical indicators (e.g., childbirth references in postpartum contexts or explicit mentions of hallucinations), the qualitative review suggests that correct predictions frequently occurred in tweets without explicit subtype keywords. In many such cases, models relied on broader contextual patterns such as mood instability, racing thoughts, or cognitive disorganization. Moreover, several misclassifications occurred in tweets with nonspecific emotional language, highlighting the inherent ambiguity of tweet-level annotations. These observations suggest that the performance improvements of fine-tuned encoders likely reflect improved contextual modeling rather than simple keyword matching, although future work could further evaluate robustness through systematic keyword masking or adversarial lexical perturbations.
